# An effective citrus ripeness detection model for complex orchard scenarios

**DOI:** 10.3389/fpls.2026.1911329

**Published:** 2026-07-24

**Authors:** Qiong Zhang, YiLiu Hang, YuanZhi Zhang, DePeng Gao, ShenTao Wang

**Affiliations:** 1 School of YonYou Digital and Intelligence, Nantong Institute of Technology, Nantong, China; 2 School of Marine Sciences, Nanjing University of Information Science and Technology, Nanjing, China; 3 Faculty of Social Science, Chinese University of Hong Kong, Hong Kong, Hong Kong SAR, China

**Keywords:** citrus ripeness, collaborative attention mechanism, dynamic depth-separable convolutions, multi-loss function, object detection

## Abstract

**Introduction:**

To address the challenges faced in detecting citrus ripeness in orchard environment, such as leaf obstructions, overlapping fruits, uneven illumination and abundant small targets, we propose an effective citrus ripeness detection model that integrates dynamic depth-separable convolutions and collaborative attention (DC-YOLO).

**Methods:**

(1) It replaces standard convolutions with dynamic depth-separable convolutions to reduce computational complexity and model parameters, while improving feature extraction adaptability. (2) It integrates a collaborative attention mechanism consisting of coordinate attention, cross-scale attention, and dual-feature multi-head self-attention. This module alleviates semantic conflicts during feature fusion and strengthens the representation of multi-scale target features and spatial localization capability. (3) A multi-loss function combining localization, classification, ripeness assessment, and occlusion awareness is adopted to meet the demands of fine-grained classification and occlusion-aware detection for citrus ripeness.

**Results:**

Experimental results demonstrate that for the citrus ripeness detection task, our model achieves a precision of 0.980, a recall of 0.965, an F1-score of 0.972, an mAP50 of 0.975 and an mAP50:95 of 0.769. Compared with the baseline model, its parameters and computational complexity are reduced by 12.79\% and 18.28\%, respectively. The inference FPS reaches 93.42, achieving a satisfactory result between detection accuracy and real-time performance.

**Discussion:**

Although our method meets the need for real-time detection of citrus ripeness in orchards, there are still challenges regarding the deployment of edge devices and the diversity of citrus data samples. Code is available at \url{https://github.com/zhangq0601/dcyolo}.

## 1 Introduction

Citrus fruits are among the most widely cultivated fruit crops in the world and play a vital role in agricultural economic development and food security. With the expansion of cultivation and advances in precision agriculture, efficient and accurate detection of citrus crops has become a key requirement. Object detection technology (Kaur and Singh, 2023; Yang et al., 2025; Zhao et al., 2026) provides a non-destructive, large-scale and real-time solution for citrus detection (Wang et al., 2025), which is crucial for improving production efficiency, reducing post-harvest losses and driving the digital transformation of agriculture (Yu et al., 2025). However, citrus object detection faces significant challenges due to the complex orchard environment and the specific growth characteristics of citrus fruits, particularly for ripeness grading (Kaur and Kumar, 2026). Conditions in natural orchards include uneven illumination, fruit and branch occlusion, and environmental disturbances. Furthermore, the gradual changes in fruit ripeness and variations between different varieties also make detection more difficult. Accurate ripeness detection is crucial for timely harvesting and quality grading, as it is closely linked to market value. Traditional manual methods are time-consuming, labor-intensive, and inaccurate — they depend on subjective judgments of peel color, leading to high misclassification rates. These methods also cannot realize non-destructive detection, thus causing substantial economic losses. Early citrus object detection primarily relied on traditional machine vision methods such as color thresholding (Guo et al., 2024) and edge detection (Wang et al., 2024). These methods depended on manual feature extraction and used peel color to determine ripeness grades. However, these methods are not well-suited to complex environments, have low accuracy. Attempts to combine electronic nose technology with color parameters are both cumbersome and unsuitable for large-scale field testing. With the advancement of deep learning, its end-to-end feature learning capabilities have made it the mainstream method for citrus object detection, overcoming the limitations of traditional methods. Various deep learning algorithms, such as Faster R-CNN (Dong et al., 2024), the YOLO series (Yue et al., 2024; Liao et al., 2025; Lin et al., 2024), SSD (Wang et al., 2023a), and RT-DETR (Zhao et al., 2024) have been applied to the task of detecting citrus fruit ripeness. For example, El Akrouchi et al. (2025) and Guo et al. (2024) proposed an intelligent citrus detection method based on the R-CNN algorithm. This method offers high accuracy and is well-suited for complex orchard environments with occlusions and dense fruit clusters. Wang et al. (2023a) and Agarwal and Bhargava (2024) proposed a citrus detection method based on a lightweight SSD algorithm. This method is lightweight and suitable for deployment on edge devices. Qiu et al. (2022); Wang et al. (2024); Feng et al. (2025), and Zeng et al. (2025) proposed a citrus detection method based on an improved YOLO model. This method offers fast detection speeds and is well-suited for edge deployment, making it ideal for tasks such as real-time orchard detection and drone inspections. Huang et al. (2025a); Wu et al. (2025), and Tang et al. (2025) proposed a method for detecting the ripeness of citrus fruits based on an improved RT-DETR model. This method employs real-time end-to-end Transformer-based object detection to rapidly detect citrus fruits, offering both high speed and high accuracy. At the same time, researchers have integrated computer vision methods—such as attention mechanisms (Chen et al., 2022; Ahmad et al., 2026; Wang et al., 2026a), multimodal approaches (Wang et al., 2026b; Chakraborty and Deka 2025), and transfer learning (Raza et al., 2025; Li et al., 2023)—into practical engineering applications to address the diverse requirements of different scenarios related to citrus ripeness, including tasks such as lightweight processing, high precision, and small-sample analysis. Despite the above achievements, practical citrus ripeness detection still faces several challenges: limited generalization across diverse citrus varieties and orchard environments, low detection accuracy for small and occluded fruits, the contradiction between model accuracy and lightweight deployment for field devices, and insufficient consideration of environmental interference in ripeness evaluation. In response to the aforementioned issues, we propose an effective model for detecting citrus ripeness that integrates dynamic depth-separable convolutions and collaborative attention. The contributions of this paper are as follows.

- We propose a dynamic depth-separable convolutional network. By improving the existing CBS module and adding a dual-attention mechanism that integrates both channel and spatial attention, the network is better able to enhance important features while suppressing irrelevant information.
- We design a collaborative attention mechanism to address the high proportion of small objects in citrus detection. We add a dedicated small-object detection head branch connected to the network’s higher-resolution feature layers, and by incorporating a coordinate-based attention mechanism, we improved the model’s detection accuracy for small objects.
- We propose a hybrid loss function. To enable the model to adapt the specific characteristics of citrus detection scenarios, we design a specialized loss function that comprehensively considers three aspects: target localization, ripeness classification, and ripeness assessment.

## 2 Materials and methods

### 2.1 Dataset construction and preprocessing

#### 2.1.1 Dataset construction

This citrus ripeness dataset was collected independently from a local citrus orchard. Close-up samples of selected fruits were captured using a standardized handheld photography method to document color differences between the peels of unripe and ripe citrus fruits. All fruits were annotated with bounding boxes in YOLO format across five ripeness categories. After filtering out invalid samples, a final set of 430 raw citrus fruit images was obtained and manually annotated according to five maturity levels: Green Fruit, Color Transition, Early Ripe, Moderately Ripe, and Fully Ripe, as shown in **Table 1**. Split the original dataset into a 7:2:1 ratio, and to enhance model robustness, data augmentation techniques including rotation, flipping, cropping, color jittering, and mosaic were applied to expand the dataset to 4,300 images. The dataset was then split into a training set (3,010 images), a validation set (860 images), and a test set (430 images) for model training and preliminary evaluation. Since there is no linear relationship between the stages of citrus ripeness and changes in peel color. When the proportion of green peel is less than 50%, the fruit is still considered unripe, and when the proportion of yellow peel exceeds 50%, the fruit’s ripening accelerates, therefore, we use a non-uniform ripeness rating scale when defining ripeness grades. Additionally, images of orchard scenes were collected, capturing occlusion, variable lighting, and fruits at various scales. They were captured from multiple angles—including straight-on, top-down, and side views—at distances ranging from 0.5 to 3 meters, and include complex scenes such as fruit and leaf occlusion, fruit overlap, and uneven lighting.

**Table 1:** Standards for classifying citrus ripeness.

Maturity rating	Tags	Tag name	Number of images	Physical characteristics	Discrete score	Regression score
Green Fruit	0	green_ripe(g)	72	The fruit is entirely green, with no yellow or orange patches on the skin or yellow and orange account for less than 20% or is on the small side.	0.0	[0.0, 0.2)
Color Transition	1	color_transition(ct)	128	The fruit is a mix of green and yellow, with yellow or orange areas accounting less than 50% of its surface.	0.5	[0.2, 0.5)
Early Ripe	2	primary_ripe(p)	98	The fruit is a mix of yellow and orange, with the yellow and orange areas accounting for 50% to 70% of the fruit, and the fruit is plump.	0.7	[0.5, 0.7)
Moderately Ripe	3	middle_ripe(m)	86	The fruit is predominantly orange, with the yellow and orange areas accounting for 70% to 90% of the surface, and the skin has a distinct texture.	0.8	[0.7, 0.9)
Fully Ripe	4	complete_ripe(cr)	46	The fruit is entirely orange or orange-red in color, plump in shape, with a glossy skin, and possesses the typical flavor of citrus fruits.	1.0	[0.9, 1.0]

#### 2.1.2 Data preprocessing

Data was augmented using a combination of geometric transformations, color enhancement, and mosaic blending, as described below.

**Figure 1:**
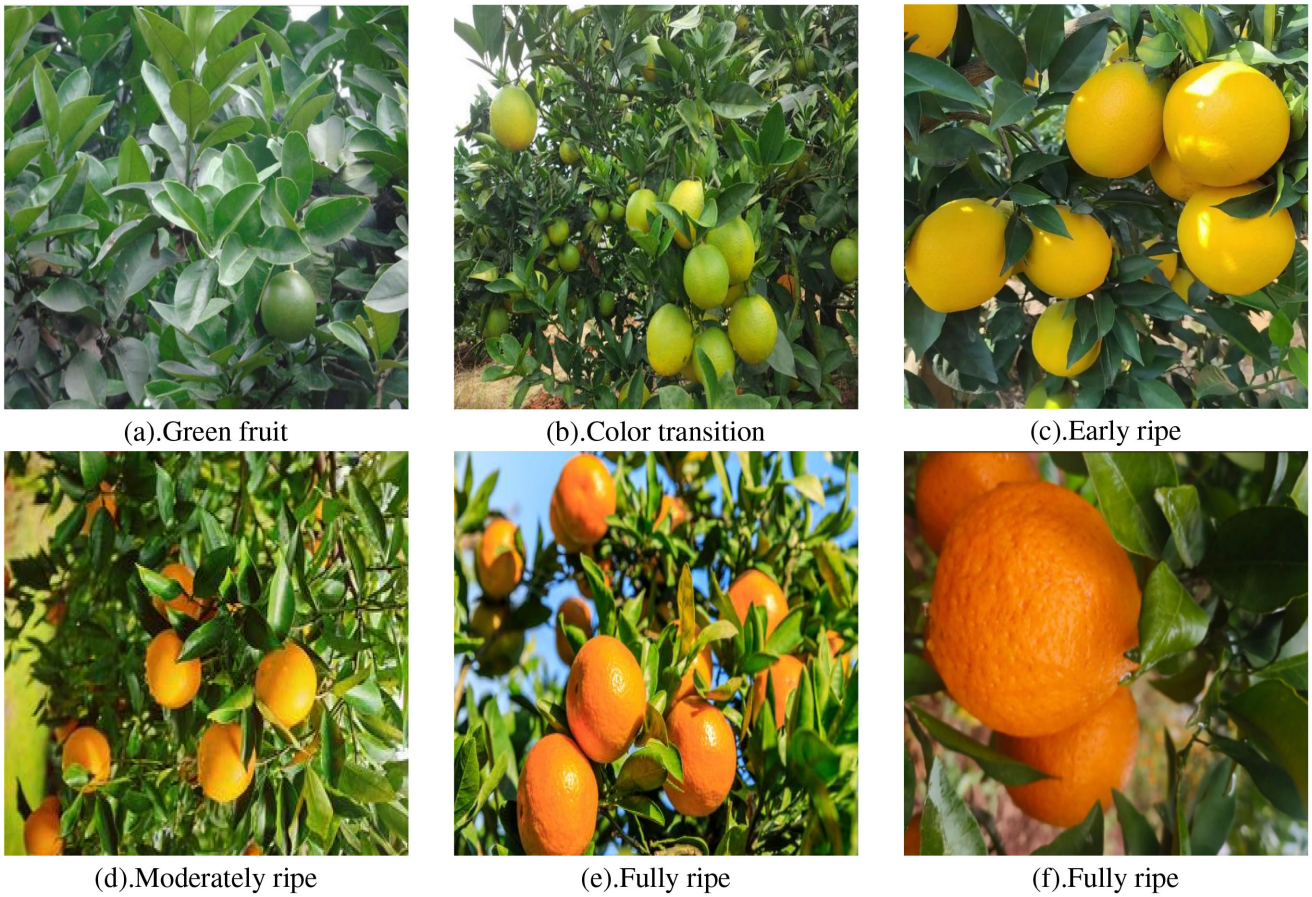
Sample data from the citrus ripeness dataset. The samples include conditions such as sunny, cloudy, and rainy days, covering the primary scenarios and variety types typically observed in orchard inspections. **(a)** Green fruit, **(b)** Color transition, **(c)** Early ripe, **(d)** Moderately ripe, and **(e, f)** Fully ripe.

1. Geometric transformations: random rotation (-45° to 45°), horizontal/vertical flipping, random cropping (0.7× to 1.0×), and scaling (0.8× to 1.2×) to simulate different shooting angles and distances.
2. Color enhancement: Randomly adjust brightness, contrast, and saturation (0.7–1.3 times), and add Gaussian and salt-and-pepper noise to simulate different lighting conditions.
3. Mosaic blending: Four images are randomly selected and combined to generate a new image, improving the model’s ability to detect small and densely clustered objects. A subset of the dataset is shown in **Figure 1**, covering different levels of ripeness, lighting conditions, occlusion levels, and varietal characteristics.

### 2.2 Overall network architecture

**Figure 2:**
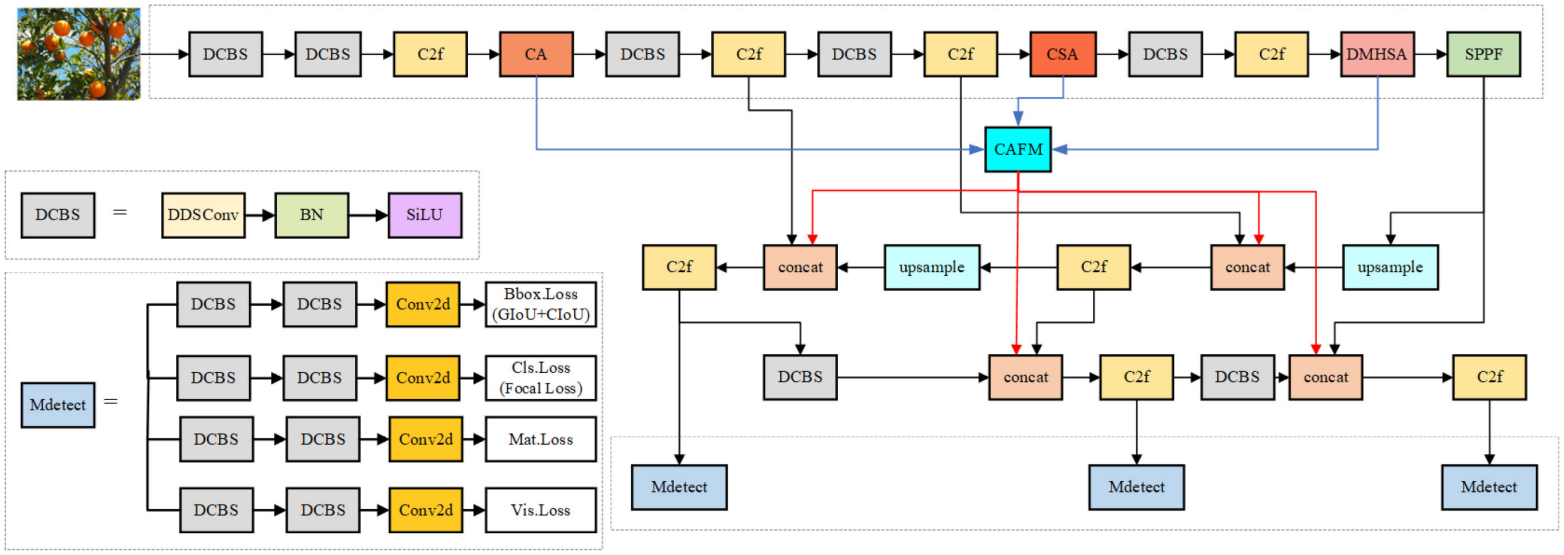
Overall network architecture. At the core of the DCBS module lies our proposed dynamic depth-separable convolutions, combined with batch normalization and the SiLU activation function. Coordinate Attention (CA) Hou et al. (2021), Cross-scale Attention (CSA) Wang et al. (2023b), Dual-feature Multihead Self-attention (DMHSA), and the Cross-layer Collaborative Fusion Module (CAFM). The black lines represent the information flow of the YOLOv8 model, the blue lines represent the collaborative attention information flow, and the red lines represent the cross-layer collaborative fusion information flow.

To address issues such as leaf occlusion, fruit overlap, uneven lighting, and a large number of small targets encountered during citrus ripeness detection, our proposed DC-YOLO method, which is different from the empirical module-stacking strategy of existing improved YOLO models, follows a systematic and hierarchical optimization logic. Dynamic depth-separable convolutions enable adaptive and lightweight low-level feature extraction. A collaborative attention mechanism addresses the representation of multiscale and occlusion features at the mid-level. Multiple loss functions provide high-level, fine-grained supervision for progressive ripeness classification. These modules correspond to feature compression, feature enhancement, and optimization constraints, respectively, and together form a complementary and theoretically rigorous holistic modeling framework. The method we propose uses YOLOv8 as a baseline. In the baseline model, the CBS module consists of convolutional layers (Conv), batch normalization (BN), and SiLU, and is responsible for channel transformation and down-sampling of feature maps (Varghese and Sambath, 2024). The CBS employs standard convolutional neural networks to compute features. Although standard convolutions can effectively extract spatial and channel features from citrus images, they require simultaneous operations across both spatial and channel dimensions. This results in a high overall computational load and lengthy inference times, making it difficult to meet the low-latency requirements of real-time citrus detection. Therefore, our method proposes a dynamic depth-separable convolution (DDSC) to replace the standard convolutions in the CBS module of YOLOv8. During citrus inspection, down-sampled features focus on semantic information such as color and texture related to fruit ripeness, while up-sampled features emphasize spatial location and edge contour details. Directly concatenating these two types of features can lead to semantic conflicts, thereby reducing the model’s accuracy in distinguishing between color changes, moderately ripe fruits, and unripe fruits. To address key challenges in citrus ripeness detection, such as leaf occlusion, multi-scale fruits, and fine-grained classification, we propose a collaborative attention mechanism. Rather than simply stacking multiple attention modules, this approach employs a “staged feature extraction + cross-layer collaborative fusion” strategy to achieve complementary functionalities in spatial localization, multi-scale completion, and semantic differentiation. In addition, the YOLOv8 loss function primarily focuses on bounding box regression and object classification. However, the traditional loss is ineffective for assessing citrus fruit ripeness. Therefore, our method comprehensively considers the three aspects of citrus fruit localization, classification, and ripeness, and designs a new loss function that specifically includes a ripeness regression branch. Given the shortcomings of YOLOv8 in citrus fruit detection described above, we redesigned the YOLOv8 network architecture by incorporating dynamic depth-separable convolutions and collaborative attention. The overall network architecture is shown in **Figure 2**.

### 2.3 Dynamic depth-separable convolutions

In citrus ripeness detection, fruits are often obscured by leaves, resulting in uneven feature distribution. The fixed weights of standard convolutions cannot distinguish between the features of the fruit and those of the obscuring leaves, which can easily lead to overfitting. While traditional deep separable convolutions are lightweight, their fixed convolutional kernels still cannot adaptively adjust feature weights, limiting their feature extraction capabilities in occlusion scenarios. Therefore, we propose a network architecture based on dynamically deep separable convolutions, which combines the advantages of adaptive feature representation in dynamic convolutions (Chen et al., 2020) with the lightweight benefits of deep separable convolutions (Chollet, 2017). The proposed dynamic depth-separable convolution (DDSC) builds upon the lightweight depthwise separable convolution skeleton from MobileNet series, and incorporates channel weighting ideas inspired by SE attention, but it is different from two related structures. Traditional dynamic convolution constructs multiple parallel convolution kernels and allocates routing weights for different samples, while DDSC does not contain a kernel bank. Its dynamic property originates from sample-adaptive channel modulation applied directly to depthwise convolution kernels before spatial feature extraction. For each input feature map, global average pooling captures content-specific channel statistics, and a lightweight MLP produces input-dependent channel weight *α_c_*. The static depthwise kernel is multiplied by *α_c_*to generate a dynamic kernel $W_{dw}^{dyn}$, which is used for subsequent spatial convolution. For occluded citrus samples, channels corresponding to leaf background features obtain low weights and are suppressed during convolution, while fruit-related channels are strengthened. In contrast, the standard SE mechanism only rescales feature maps after convolution as post-processing, with fixed convolution kernels throughout forward propagation. The DDSC modulates convolution kernels in advance, realizing dynamic feature screening at the feature extraction stage rather than filtering redundant information afterward, which brings better discrimination between citrus fruit and occluded leaf features. It also differs from spatial kernel attention, GhostConv (Han et al., 2020) and static MobileNet blocks, as none of these designs realize kernel-level adaptive channel modulation before convolution. The complete process is as follows.

#### 2.3.1 Global average pooling

By modeling channel features of the input feature maps using a lightweight squeeze-and-excitation (SE) module (Hu et al., 2018), channel attention vectors are generated to enable adaptive allocation of importance across different channels.

1. Global average pooling(GAP) compresses spatial features, as shown in Equation 1.

(1) $$z_{c}=\frac{1}{H\times W}\sum_{i=1}^{H}\sum_{j=1}^{W}X(i,j,c)$$

*z_c_*represents the feature vector of the *c* th input channel after global average pooling.

2. Two fully connected layers and an activation function generate attention weights, as shown in Equations 2 and 3.

(2) $$\hat{z}=\text{LeakyReLU}(W_{1}z+b_{1})$$

(3) $$\alpha_{c}=\text{Sigmoid}(W_{2}\hat{z}+b_{2})$$

LeakyReLU preserves the baseline response across all channels. Sigmoid normalizes the weights to the range [0,1], allowing for precise allocation of importance across different channels. For example, by suppressing the weights of channels obscured by leaves. $\hat{z}$ denotes the intermediate feature vector after activation by LeakyReLU. $W_{1}$ and $W_{2}$ denote the weight matrices of the two fully connected layers, $W_{1}\in R^{C_{in}/r\times C_{in}}$, $W_{2}\in R^{C_{in}\times C_{in}/r}$, respectively, where r is the dimension reduction factor, set to 16. $z={\left[z_{1},z_{2},\ldots ,z_{C_{in}}\right]}^{T}\in R^{C_{in}}$, $b_{1}$ and $b_{2}$ denote the corresponding bias terms of the two fully connected layers. $\alpha_{c}$ denotes the attention weight corresponding to the c th channel.

#### 2.3.2 Dynamic deep convolutional kernel construction

Fuse the convolutional kernel with the channel attention vector to generate a dynamic convolutional kernel, as shown in Equation 4.

(4) $$W_{dw}^{dyn}(k_{1},k_{2},c)=W_{dw}(k_{1},k_{2},c)\cdot \alpha_{c}$$

$W_{dw}^{dyn}$ denotes the weighted dynamic depth convolution kernel, $W_{dw}$ denotes the static depth convolution kernel, $\left(k_{1},k_{2}\right)$ denotes the spatial index of the depth convolution kernel, $k_{1},k_{2}\in \{0,1,2\}$ and c denotes the input channel index.

#### 2.3.3 Dynamic depth-separable convolutional computation

Perform spatial convolution using dynamic depth convolution kernels, then fuse cross-channel information through pixel-wise convolution.

1. Dynamic depth convolution for spatial feature extraction, as shown in Equation 5.

(5) $$X_{dw}^{dyn}(i,j,c)=\sum_{k_{1}=1}^{K}\sum_{k_{2}=1}^{K}W_{dw}^{dyn}(k_{1},k_{2},c)\cdot X(i+k_{1},j+k_{2},c)$$

2. Pointwise convolution for channel feature fusion, as shown in Equation 6.

(6) $$Y(i,j,c_{out})=\sum_{c=1}^{C_{in}}W_{pw}(1,1,c,c_{out})\cdot X_{dw}^{dyn}(i,j,c)+b_{pw}(c_{out})$$

$X_{dw}^{dyn}$ represents the output feature map of the dynamic depth convolution, $W_{pw}$ represents the weights of the 1×1 convolution kernel at each pixel, $c_{out}$ represents the output channel index of the pixel-wise convolution, and $b_{pw}$ represents the bias term of the pixel-wise convolution.

**Figure 3:**
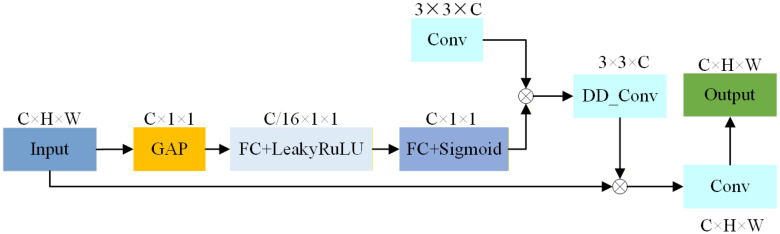
Dynamic Depth Separable Convolution (DDSC) architecture; DD Conv denotes Dynamic Depth Convolution.

By dynamically adjusting the weights of the convolutional kernels, the model can adaptively focus on the effective features of citrus fruits, enhancing feature extraction capabilities in occlusion scenarios while maintaining a lightweight architecture, as shown in **Figure 3**.

### 2.4 Collaborative attention mechanism

The collaborative attention mechanism consists of coordinate attention (CA), cross-scale attention (CSA), and dual-feature multi-head self-attention (DMHSA), deployed respectively in the shallow, middle, and deep layers of the backbone. Information is then exchanged through a cross-layer collaborative fusion module before being fed into the neck for multi-scale feature fusion, thereby fundamentally alleviating the semantic conflict issues caused by concatenation operations.

#### 2.4.1 Coordinate attention mechanism

**Figure 4:**
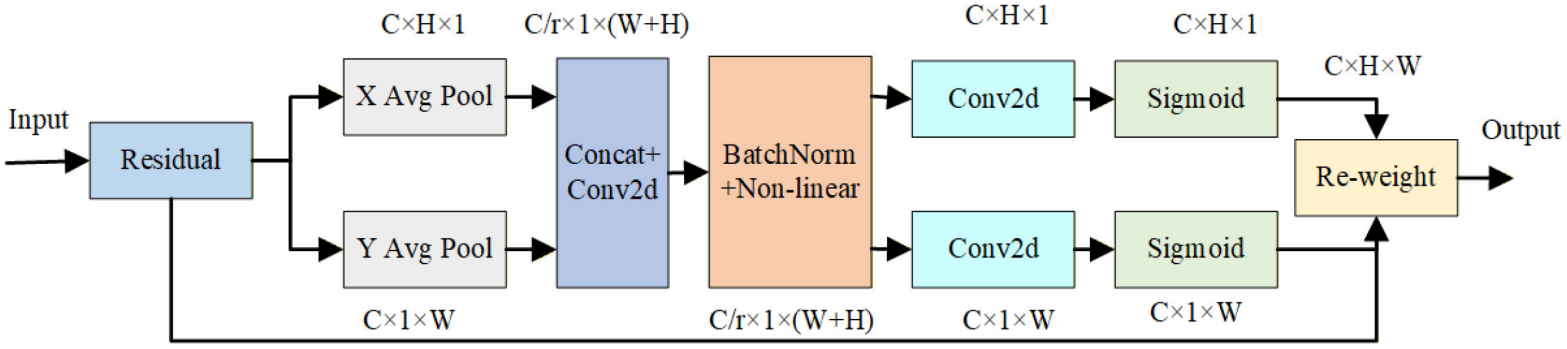
Structural diagram of the coordinate attention mechanism. Coordinate attention splits global average pooling into height-wise and width-wise perceptual pooling, generating horizontal and vertical feature representations, respectively. These are then passed through fully connected layers and activation functions to generate position-sensitive attention weights, which are ultimately used to weight and enhance the input feature maps, enabling the model to focus more on the spatial contours and positional information of the fruit.

Coordinate attention captures long-range spatial dependencies while preserving precise positional information by decomposing channel attention (Wang et al., 2020) into one-dimensional feature encodings along horizontal and vertical directions. It offers the advantages of being lightweight, efficient, and highly position-aware. Given that the shallow layers of the YOLOv8 backbone feature high resolution and rich spatial information but lack semantic richness, we deploy the coordinate attention mechanism after the output of the third layer of the backbone to precisely localize citrus fruit regions and suppress background noise interference. The computational process of the coordinate attention mechanism is as follows, as shown in **Figure 4**.

#### 2.4.2 Cross-scale attention

In citrus ripeness detection, due to significant variations in fruit size across different ripeness stages, as well as issues such as fruit overlap caused by dense planting and partial occlusion by leaves, a cross-scale attention mechanism has been proposed. This approach establishes correlation dependencies between features at different scales, enabling precise detection of multi-scale targets and feature enhancement. The computation of cross-scale attention is primarily divided into four steps. Let the input feature map be $\in R^{H\times W\times C}$, where H and W denote the height and width of the feature map, respectively, and C denotes the number of channels. The output enhanced feature map is $Y\in R^{H\times W\times C}$. The computational process for cross-scale attention is as follows.

1. Multi-path parallel feature extraction. Designing feature extraction for three different receptive fields. Path 1: Using a convolutional kernel of 1×1, this approach preserves the original image dimensions and fine-grained features, focusing on the edges and textural features of small, unripe citrus fruits. The calculation process is shown in Equation 7.

(7) $$X_{1}={\text{Conv}}_{1\times 1}(X)=W_{1}*X+b_{1}$$

where $W_{1}\in R^{1\times 1\times C\times C}$ represents the weights of the 1×1 convolution kernel, $b_{1}\in R^{C}$ represents the bias term, * denotes the convolution operation, and $X_{1}\in R^{H\times W\times C}$ represents the output.

Path 2: By using a 3×3 convolutional kernel, this method enhances the semantic expressiveness of features while maintaining the same scale, making it suitable for feature extraction from medium-sized semi-ripe citrus fruits. The calculation process is shown in Equation 8.

(8) $$X_{2}={\text{Conv}}_{3\times 3}(X)=W_{2}*X+b_{2}$$

$W_{2}\in R^{3\times 3\times C\times C}$ represents the weights of the 3×3 convolutional kernel, $b_{2}\in R^{C}$ represents the bias term, and $X_{2}\in R^{H\times W\times C}$ represents the output.

Path 3: Using a combination of “3×3 down-sampling convolution ( s=2,p=1) + transposed up-sampling convolution ( s=2,p=1,o=1)”, we reduce the spatial dimension to enhance global semantic features for adapting to large, mature citrus fruits, and then restore the original scale to ensure feature alignment. To eliminate alignment error, we configure output padding *o* = 1 for transposed convolution to strictly recover the original spatial dimension, removing inherent pixel offset mathematically. Second, a parallel 1×1 convolution branch is introduced to reserve undistorted shallow small-target features, which compensates spatial feature drift generated in the down–up reconstruction path. The calculation process is shown in Equations 9 and 10.

(9) $$X_{2d}={\text{Conv}}_{3\times 3}(X)=W_{3}*X+b_{3}\;(X_{2d}\in R^{(H/2)\times (W/2)\times C})$$

(10) $$X_{3}={\text{DeConv}}_{3\times 3}(X_{2d})=W_{4}*X_{2d}+b_{4}$$

Where $W_{3}\in R^{3\times 3\times C\times C}$ is the weight of the down-sampling convolution kernel(s=1,p=1), $W_{4}\in R^{3\times 3\times C\times C}$ is the weight of the transposed convolution kernel, $b_{3},b_{4}\in R^{C}$ is the bias term, and the output is $X_{3}\in R^{H\times W\times C}$.

2. Cross-path feature fusion and global information extraction. The output features from the three paths are summed element-wise to obtain preliminary fused features. GAP is then applied to extract global semantic information, which serves as a guide for generating attention weights. The calculation process is shown in Equations 11 and 12.

(11) $$X_{\text{sum}}=X_{1}+X_{2}+X_{3}\;(X_{\text{sum}}\in R^{H\times W\times C})$$

(12) $$X_{\text{gap}}=\text{GAP}(X_{\text{sum}})=\frac{1}{H\times W}\sum_{i=1}^{H}\sum_{j=1}^{W}X_{\text{sum}}(i,j,:)\;(X_{\text{gap}}\in R^{C})$$

3. Generation of cross-path attention weights. By generating channel-level attention vectors through a compression and activation structure, the model dynamically adjusts the relative importance of features across the three paths, enabling cross-path attention interaction.

Path1: Channel compression. Reduce the number of channels in the global features $X_{\text{gap}}$ to *C/r* via a 1×1 convolution, where r is the compression ratio and in the experiments, r is set to 4 to balance accuracy and efficiency. The calculation process is shown in Equation 13.

(13) $$X_{\text{compress}}={\text{Conv}}_{1\times 1}(X_{\text{gap}})=W_{5}\cdot X_{\text{gap}}+b_{5}\;(X_{\text{compress}}\in R^{C/r})$$

Path2: Nonlinear activation. The GeLU activation function is used to introduce nonlinearity, enhancing the expressive power of the attention vectors. The calculation process is shown in Equation 14.

(14) $$X_{\text{act}}=\text{GeLU}(X_{\text{compress}})=X_{\text{compress}}\cdot \text{Φ}(X_{\text{compress}})$$

Φ(·) is the cumulative distribution function of the standard normal distribution.

Path3: Channel restoration and weight normalization. The number of channels is restored via 1×1 convolutions, and the weights are then compressed to the range [0,1] using a sigmoid function to obtain the cross-path attention vector $\;\alpha \in R^{C}$, as shown in Equation 15.

(15) $$\alpha =\text{Sigmoid}({\text{Conv}}_{1\times 1}(X_{\text{act}}))=\text{Sigmoid}(W_{6}\cdot X_{\text{act}}+b_{6})$$

4. Attention-weighted feature aggregation. Apply the attention vectors *α* to the features of the three paths to achieve dynamic weighting, then perform channel fusion via 1×1 convolutions to produce the final enhanced features, as shown in Equations 16–18.

(16) $$X_{1^{\prime }}=\alpha ⨀X_{1},\;X_{2^{\prime }}=\alpha ⨀X_{2},\;X_{3^{\prime }}=\alpha ⨀X_{3}$$

(17) $$X_{\text{concat}}=\text{Concat}(X_{1^{\prime }},X_{2^{\prime }},X_{3^{\prime }})\;(X_{\text{concat}}\in R^{H\times W\times 3C})$$

(18) $$Y=\text{GeLU}({\text{Conv}}_{1\times 1}(X_{\text{concat}}))=\text{GeLU}(W_{7}*X_{\text{concat}}+b_{7})$$

⨀ denotes element-wise multiplication, concat(·) denotes channel-wise concatenation, $W_{7}\in R^{1\times 1\times 3C\times C}$ represents the weights of the aggregated convolution kernel, and $b_{7}\in R^{C}$ represents the bias term.

#### 2.4.3 Dual-feature multi-head self-attention

The dual-feature multi-head attention mechanism builds upon the Transformer’s multi-head self-attention by integrating features extracted via channel attention and spatial attention. Through channel filtering and spatial focusing, it eliminates redundant information, providing high-quality input for the multi-head self-attention mechanism and reducing the computational overhead of global modeling. Assuming the input feature map is $X\in R^{H\times W\times C}$, and the output enhanced feature is $Y_{\text{final}}\in R^{H\times W\times C}$. The computational process for dual-feature multi-head self-attention is as follows.

1. Dual-attention feature extraction. Dual-attention feature extraction primarily involves the extraction of channel features and spatial features.

Channel attention: Enhance channels related to citrus ripeness and suppress background channels.

Global feature compression: *X*_gap_ = GAP(*X*), *X*_gmp_ = GMP(*X*), GAP stands for global average pooling, and GMP stands for global max pooling.

Weight generation: *X*_cat_ = Concat(*X*_gap_*,X*_gmp_).

The channel weights are obtained through the fully connected layer *α* ∈ *R^C^*.

Channel weighting: *X*_ca_ = *α* ⨀ *X*_cat_, ⨀ is expressed as element-wise multiplication, *X*_ca_ ∈ *R^H^*^×^*^W^*^×^*^C^*.

Spatial attention: Focus on the citrus fruit area and blur the background.

Spatial feature compression: *X*_avg_ = AvgPool(*X*_ca_), *X*_max_ = MaxPool(*X*_ca_), AvgPool stands for average pooling, and MaxPool stands for max pooling.

Weight generation: $\;X_{\text{cat}}^{s}=\text{Concat}(X_{\text{avg}},X_{\text{max}})$, the spatial weights $\beta \in R^{H\times W}$ are obtained using $Conv_{7\times 7}$ and Sigmoid.

Spatial weighting: $\;X_{\text{dual}}=\beta ⊗X_{\text{ca}}$, ⊗ represents matrix multiplication, $X_{\text{dual}}\in R^{H\times W\times C}$.

2. Multi-head self-attention with global integration. Based on the refined data *X*_dual_, model the relationship between citrus fruits and the overall scene to optimize the semantic differentiation of ripeness.

Feature flattening and mapping: $X_{\text{seq}}=\text{Reshape}(X_{\text{dual}})\in R^{N\times C},N=H\times W$, $Q=W_{q}\cdot X_{\text{seq}}$, $K=W_{k}\cdot X_{\text{seq}}$, $V=W_{v}\cdot X_{\text{seq}}$.

Attention calculation for multi-head networks: Break down *Q/K/V* into h = 8 headers, individual header:

${\text{Attn}}_{i}=\text{Softmax}(\frac{Q_{i}\cdot K_{i}^{T}}{\sqrt{d_{k}/h}})\cdot V_{i},({\text{Attn}}_{i}\in R^{N\times d_{k}/h})$Integration and recovery: Attn_concat_ = Concat(Attn_1_*,…*,Attn*_h_*). Using *X*_mhsa_ = *W_o_*· Attn_concat_ and Reshape, we obtain *Y* ∈ *R^H^*^×^*^W^*^×^*^C^*.

**Figure 5:**
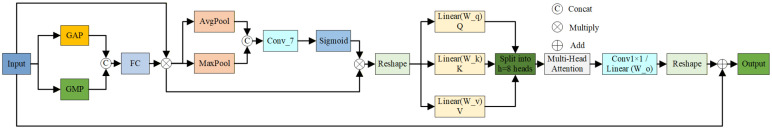
Dual-feature multi-head self-attention mechanism. This module first extracts channel attention and spatial attention separately to perform weighted feature refinement. It then maps the refined features to query, key, and value vectors, splitting them into multiple attention heads to compute global dependencies in parallel. Finally, it combines the original features with the fused features via a residual connection to prevent gradient vanishing while improving the classification accuracy of the mature model.

Finally, to prevent gradient vanishing, the original features are combined with the fused features: *Y*_final_ = *Y* + *X*. The workflow of the dual-feature multi-head self-attention mechanism is shown in **Figure 5**.

#### 2.4.4 Cross-layer attention fusion module

To achieve functional synergy among coordinate attention, cross-scale attention, and dual-feature multihead self-attention, and to avoid the issue of feature fragmentation caused by these three mechanisms operating in isolation, our method designs a cross-layer attention collaboration and fusion module. This module dynamically fuses the output features of the three attention mechanisms using weighted averaging to generate synergistically enhanced features, which are then fed into the concatenation operation of the Neck. The specific process and calculation formula are as follows.

1. Alignment of feature dimensions. Set the output features of coordinate-based attention to $F_{ca}\in R^{H_{1}\times W_{1}\times C_{1}}$, which is shallow-layer output and high resolution. The output features of cross-scale attention to $F_{cmsa}\in R^{H_{2}\times W_{2}\times C_{2}}$, which is mid-layer output and medium resolution. The output features of dual-feature multi-head self-attention to $F_{mhsa}\in R^{H_{3}\times W_{3}\times C_{3}}$, which is deep-layer output and low resolution. Because the spatial dimensions and channel counts of the three features differ, they cannot be directly fused. Therefore, they must be size-aligned by uniformly down-sampling $F_{ca}$ and $F_{cmsa}$ to the spatial dimension of $F_{mhsa}(H_{3}\times W_{3})$, while simultaneously using 1×1 convolution to unify their channel counts to C, resulting in aligned features. The calculation formula is as shown in Equations 19–21.

(19) $$F_{ca}^{align}=\text{ConvDown}(F_{ca},k=2\times 2,s=2)$$

(20) $$F_{cmsa}^{align}=\text{ConvDown}(F_{cmsa},k=2\times 2,s=2)$$

(21) $$F_{mhsa}^{align}=F_{mhsa}$$

ConvDown(x) denotes a down-sampling convolution with a stride of 2 and a kernel size of 2×2, used to reduce the dimensionality of the feature space, where k is the kernel size and s is the stride. $F_{ca}^{align}$, $F_{cmsa}^{align}$ and $F_{mhsa}^{align}$ are the outputs of the three types of attention features after alignment, all with dimensions of $H_{3}\times W_{3}\times C$.

2. Cooperative weight generation. To achieve adaptive fusion of the three types of attention features, dynamic collaborative weights must be generated to adjust the relative importance of each feature. First, GAP is applied to each of the three aligned features to extract channel-level global feature vectors and eliminate the influence of spatial dimensions.

The calculation process is shown in Equations 22–24.

(22) $$f_{ca}=\text{GAP}(F_{ca}^{align})=\frac{1}{H_{3}\times W_{3}}\sum_{i=1}^{H_{3}}\sum_{j=1}^{W_{3}}F_{ca}^{align}(i,j,:)\in R^{1\times 1\times C}$$

(23) $$f_{cmsa}=\text{GAP}(F_{cmsa}^{align})=\frac{1}{H_{3}\times W_{3}}\sum_{i=1}^{H_{3}}\sum_{j=1}^{W_{3}}F_{cmsa}^{align}(i,j,:)\in R^{1\times 1\times C}$$

(24) $$f_{mhsa}=\text{GAP}(F_{mhsa}^{align})=\frac{1}{H_{3}\times W_{3}}\sum_{i=1}^{H_{3}}\sum_{j=1}^{W_{3}}F_{mhsa}^{align}(i,j,:)\in R^{1\times 1\times C}$$

$f_{ca}$, $f_{cmsa}$ and $f_{mhsa}$represent the channel-level feature vectors obtained by applying global average pooling to the three aligned features, respectively.

The three global feature vectors are then concatenated along the channel dimension to obtain the concatenated feature vector $f_{concat}$. This vector undergoes channel compression via a 1×1 convolutional layer, and the ReLU activation function is introduced to enhance nonlinear representational capacity. Subsequently, the channel dimension is restored via a 1×1 convolutional layer, and finally, the output is normalized to the interval [0,1] via the Sigmoid activation function, generating the dynamic collaborative weight vector. The calculation process is shown in Equations 25–29.

(25) $$f_{concat}=\text{Concat}(f_{ca},f_{cmsa},f_{mhsa})\in R^{1\times 1\times 3C}$$

(26) $$f_{compress}={\text{Conv}}_{1\times 1}(f_{concat},W_{c},b_{c})\in R^{1\times 1\times C/r}$$

(27) $$f_{activate}=\text{ReLU}(f_{compress})$$

(28) $$f_{restore}={\text{Conv}}_{1\times 1}(f_{activate},W_{r},b_{r})\in R^{1\times 1\times 3C}$$

(29) $$W_{co}=\text{Sigmoid}(f_{restore})=[W_{ca},W_{cmsa},W_{mhsa}]\in R^{1\times 1\times 3C}$$

$W_{c}$ and $W_{r}$ are the weight matrices for convolution during channel compression and channel restoration, respectively, while $b_{c}$ and $b_{r}$ are the corresponding bias terms, r is the dimension-reduction coefficient, set to 16 to balance model accuracy and computational efficiency. $W_{co}$ can be decomposed into three sub-weight vectors $W_{ca}$, $W_{cmsa}$ and $W_{mhsa}$, each of dimension 1 ×1 ×C, corresponding to the weights of the coordinate attention, cross-scale attention, and dual-feature multi-head self-attention features, respectively.

(3) Weighted feature fusion. The generated dynamic collaborative weights are applied to the corresponding aligned features, and feature weighting is achieved through element-wise multiplication. The three weighted features are then summed element-wise to obtain the final collaborative enhanced feature $F_{co}$. This feature integrates the strengths of the three attention mechanisms, providing precise spatial localization information, comprehensive multi-scale features, and clear maturity-based semantic features simultaneously, as shown in Equation 30.

(30) $$F_{co}=W_{ca}⨀F_{ca}^{align}+W_{cmsa}⨀F_{cmsa}^{align}+W_{mhsa}⨀F_{mhsa}^{align}$$

**Figure 6:**
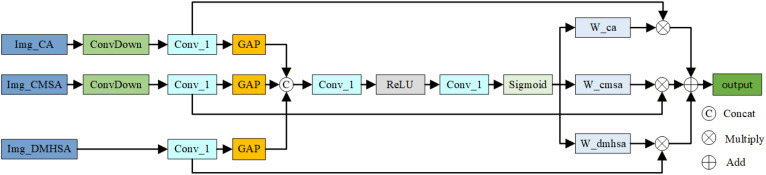
Cross-layer attention-based collaborative fusion.

⨀ denotes element-wise multiplication; $F_{co}\in R^{H_{3}\times W_{3}\times C}$ represents the final output features resulting from cross-layer collaborative fusion. By feeding these directly into the concatenation operation of the Neck—replacing the original output features of the Backbone—this approach effectively mitigates the semantic conflicts caused by directly concatenating down-sampled and up-sampled features, thereby further improving the model’s accuracy in detecting citrus ripeness. The schematic diagram of cross-layer attention-based collaborative fusion is shown **Figure 6**.

### 2.5 Multi-loss function

The appropriate use of a loss function can optimize a model’s ability to accurately detect objects. The traditional YOLOv8 loss function primarily focuses on bounding box regression and object classification. However, this approach is ineffective for assessing citrus fruit ripeness. Therefore, we comprehensively consider the three aspects of citrus fruit localization, classification, and ripeness to design a new loss function, specifically incorporating a ripeness regression branch including ripeness assessment and visibility awareness. Citrus ripeness is a gradual continuous color change rather than a rigid categorical jump. Pure discrete classification ignores the gradual transition between adjacent ripeness grades, which easily causes misclassification of borderline samples. We adopt a dual-task optimization strategy: (1) Discrete classification loss distinguishes five independent ripeness categories and provides hard category constraints; (2) Continuous regression loss predicts a continuous ripeness score, which quantifies the subtle color differences between transitional samples. These two tasks are mutually constrained: the category limits the range of regression values, and the regression score further refines the ripeness degree within the same category. Joint optimization alleviates the misclassification of transitional citrus samples, which is highly consistent with the actual gradual ripening law of citrus fruits, as shown in Equation 31.

(31) $$L=L_{loc^{\prime }}+L_{cls^{\prime }}+L_{mat}+\lambda_{vis}L_{vis}$$

$L_{loc^{\prime }}$ denotes the bounding box regression loss, $L_{cls^{\prime }}$ denotes the maturity classification loss, $L_{mat}$ denotes the maturity regression loss, $L_{vis}$ denotes the visibility-aware loss, and $\lambda_{vis}$ denotes the corresponding loss weight, which is set to 0.5, for verification results, please refer to **Supplementary Table 1** in the appendix.

The bounding box regression loss( $L_{loc}$) combines GIoU and CIoU while adjusting the aspect ratio penalty term, the calculation process is shown in Equations 32–34.

(32) $$L_{loc}=L_{GIoU}+\alpha \times L_{C_{IoU}}^{adjusted}$$

(33) $$L_{GIoU}=1-IoU+\frac{\left|C-(B\cup B^{gt})\right|}{\left|C\right|}$$

(34) $$L_{CIoU}^{adusted}=1-IoU+\frac{\rho^{2}(B,B^{gt})}{c^{2}}+a_{V}\times V$$

α denotes balance factor between GIoU loss and modified CIoU loss, fixed to 0.6. B denotes the predicted bounding box. $B^{gt}$ denotes the ground-truth bounding box. V denotes aspect ratio consistency penalty term in standard CIoU, measuring the discrepancy of width–height ratio between predicted box B and ground truth $B^{gt}$. $a_{V}$ denotes adaptive weight coefficient for the aspect ratio penalty, designed to scale V according to fruit scale in citrus datasets. For small occluded citrus targets, $a_{V}$ is elevated to strengthen aspect ratio constraint, while for large complete fruits, $a_{V}$ is reduced to avoid over-penalization. C is the smallest bounding box that contains both. ρ(x,y) is the Euclidean distance between the elements of x and y, and c is the length of the diagonal of C.

Focal Loss( $L_{cls}$) was used to address the classification loss for ripeness categories, as shown in Equation 35. In citrus ripeness detection, there are significant differences among fruits at different ripeness levels. The Focal Loss function effectively mitigates the issues caused by class imbalance by reducing the weight of easily classified samples and focusing attention on the difficult-to-classify samples. Citrus ripeness was categorized into five classes: unripe, color transition, early ripe, medium ripe, and fully ripe, with corresponding labels of 0.0, 0.5, 0.7, 0.8, and 1.0, respectively, as shown in **Table 1**.

(35) $$L_{cls}=-\sum_{i=1}^{N}\alpha_{i}{\left(1-p_{i}\right)}^{\gamma }\text{log }(p_{i})$$

$p_{i}$ represents the predicted probability that the i th sample belongs to the true class, $\alpha_{i}$ is the class balance factor, and γ is the focus parameter, set to 2.

The ripeness regression loss ( $L_{mat}$) uses a smoothed L1 loss function, as shown in Equation 36. Ripeness assessment essentially involves predicting the transition of citrus fruits from green to orange by mapping regression scores to corresponding ripeness levels, as shown in **Table 1**. When the loss error is small, the smoothed L1 loss behaves like a squared loss; when the error is large, it behaves like a standard L1 loss. This dynamic adjustment effectively prevents the problem of gradient explosion. To further improve the model’s ability to distinguish between different stages of citrus ripeness, stage weights are incorporated into the loss function, with higher weights assigned to the moderately ripe and fully ripe stages.

(36) $$L_{mat}=\sum_{i=1}^{N}W_{stage}(m_{i})\times SmoothL1(m_{i},{\hat{m}}_{i})$$

*m_i_*represents the actual maturity score, and ${\hat{m}}_{i}$represents the predicted maturity score.

Visibility-aware loss ( $L_{vis}$) adjusts the weights of the bounding box regression and classification losses by predicting the proportion of the target that is visible, as shown in Equation 37.

(37) $$L_{vis}=BCE(\hat{v},v)$$

*v* represents the ground-truth visibility score, $\hat{v}$ represents the predicted visibility score, and *BCE*(*x*) represents the binary cross-entropy loss function.

By adjusting the visibility-aware loss value, we can modify the proportion of the visible portion of the prediction target while simultaneously adjusting other losses. The formulas for the adjusted bounding box regression loss and maturity class classification loss, as shown in Equation 38.

(38) $$\begin{array}{l} L_{loc^{\prime }}=v\times L_{loc}\\ L_{cls^{\prime }}=v\times L_{cls} \end{array}$$

By modifying the loss function, we can lower the prediction requirements for occluded citrus fruits, thereby improving the prediction accuracy of the visible parts and increasing the classification accuracy of the citrus fruits.

## 3 Results

### 3.1 Experimental environment

The hardware and software configuration for this experiment is shown in **Table 2**. All models were trained and tested under the same experimental conditions to ensure the reproducibility and consistency of the results.

**Table 2:** Experimental environment configuration.

Category	Configuration details
Hardware Platform	Intel(R) Core(TM) i7-13700K CPU 3.40GHz; NVIDIA RTX 4090 24G GPU; 32G DDR4
Software Platform	Windows 11 64bit Operating System; Python 3.9.18
Deep learning frameworks	PyTorch 2.0.1; CUDA 11.7; CuDNN 8.9.2
Image Processing Library	OpenCV 4.8.0; PIL 9.5.0; Matplotlib 3.7.2
Annotation tool	LabelImg 1.8.6
Assessment Tool	Ultralytics 8.0.20; Scikit-learn 1.3.0

### 3.2 Experimental setup

Training parameters: batch size = 16, input size=512 × 512, initial learning rate = 1.0× 10^−3^, cosine smoothed learning rate decay, weight decay coefficient = 5.0× 10^−3^, optimizer is SGD with momentum and momentum = 0.937, number of epochs = 100, early stopping strategy (patience = 10), global random seed = 42; Test parameters: confidence threshold = 0.5; non-Maximum suppression (NMS) intersection ratio threshold = 0.45; Generalization validation setup: The external dataset uses a real-world field test subset consisting of 80 images to evaluate the detection performance of models trained on the training datasets across different orchards and varieties. The external dataset includes five ripeness labels: 11 images for green fruit, 16 for color transition, 20 for early ripe, 17 for moderately ripe and 16 for fully ripe.

### 3.3 Performance evaluation metrics

The evaluation criteria consist of three categories: detection performance metrics, model lightweighting metrics, and generalization performance metrics. Performance metrics: Precision (P), Recall (R), F1-score, mAP50 and mAP50:95. Model lightweighting metrics: number of parameters (Params), computational complexity (FLOPs), and frame rate (FPS). The calculation process is shown in Equations 39–42.

(39) $$Precision=\frac{TP}{TP+FP}$$

(40) $$Recall=\frac{TP}{TP+FN}$$

(41) $$F1=\frac{2\times Precision\times Recall}{Precision+Recall}$$

(42) $$mAP=\frac{1}{N}\sum_{i=1}^{N}AP_{i}$$

*TP* represents the number of true positive samples, *FP* represents the number of false positive samples, *FN* represents the number of false negative samples, *N* represents the number of maturity categories, and *AP_i_*represents the average precision for the i th maturity category.

### 3.4 Ablation experiment

To validate the rationality of the model modifications and assess their impact on improving the detection performance for citrus ripeness, we design ablation experiments. These experiments compared the YOLOv8 model with various modifications, which include replacing the CBS module with dynamic depth-separable convolution (DDSC), introducing a collaborative attention mechanism (CAM), and improving the multiloss function (MLF), as well as combinations of these improvements. The experimental results are shown at **Table 3**, calculated as the average detection values across all citrus varieties.

**Table 3:** Comparison of ablation experiment results.

Baseline	DDSC	CAM	MLF	Precision	Recall	F1	mAP50	mAP50:95
✓	×	×	×	0.920	0.901	0.910	0.881	0.673
✓	✓	×	×	0.945	0.905	0.925	0.902	0.683
✓	×	✓	×	0.936	0.931	0.933	0.913	0.715
✓	×	×	✓	0.944	0.925	0.934	0.906	0.702
✓	✓	✓	×	0.942	0.932	0.937	0.938	0.721
✓	✓	×	✓	0.947	0.935	0.941	0.943	0.728
✓	×	✓	✓	0.955	0.951	0.953	0.961	0.734
✓	✓	✓	✓	**0.980**	**0.965**	**0.972**	**0.975**	**0.769**

Bold values indicate the optimal values.

Ablation experiments verify the effectiveness of each proposed component. The baseline YOLOv8 achieves the lowest detection precision. Individual introduction of DDSC, CAM and MLF all improves model performance slightly. When the CBS module of baseline is replaced with DDSC, Precision increases by 2.72%, F1 increases by 1.65%, mAP50 increases by 2.38% and mAP50:95 increases by 1.49%. The CAM module is added in baseline, which brings a 1.74% increase in precision, a 3.33% increase in recall, a 2.53% increase in F1, a 3.63% improvement in mAP50 and a 6.24% increase in mAP50:95. The MLF module achieves a 2.61% precision improvement, a 2.66% recall improvement, a 2.64% F1 improvement, a 2.84% mAP50 improvement and a 4.31% mAP50:95 improvement. All dual-module combinations exhibit obvious synergistic effects with performance gains exceeding the simple superposition of individual modules, which bring further performance gains due to positive synergy. The full DC-YOLO integrating DDSC, CAM and MLF obtains the optimal Precision, Recall, F1, mAP50 and mAP50:95, which demonstrates that the joint optimization of adaptive feature extraction, cross-scale attention and multi-loss function can comprehensively enhance the model’s capability for fine-grained citrus ripeness detection under complex orchard environment.

### 3.5 Comparison of model performance

**Table 4** quantitatively evaluates the performance of each improved module in terms of parameter volume, computational overhead and inference speed, taking the original YOLOv8 as the baseline. The introduction of the DDSC lightweight convolution drastically reduces model parameters by 18.21% and GFLOPs by 36.46%, and raises FPS by 7.77%, which verifies its strong capability for model compression. When CAM or MLF is further embedded upon model, parameters and computation increase slightly while FPS remains far higher than the baseline. Finally, the complete DC-YOLO integrating DDSC, CAM and MLF achieves a 12.79% reduction in parameters and an 18.28% drop in GFLOPs; meanwhile, its FPS is improved by 15.92% compared with vanilla YOLOv8. The results prove that the proposed multicomponent improvement scheme realizes favorable lightweight balance, delivering faster inference with lower computational cost.

**Table 4:** Comparison of model parameter counts, computational load, and detection frame rates.

Combination	#Params(M)↓	GFLOPs↓	FPS	Parameters reduction ratio	Computational reduction ratio	Efficiency growth ratio
YOLOv8	11.26	18.54	80.59	–	–	–
YOLOv8+DDSC	9.21	11.78	86.85	18.21%	36.46%	7.77%
YOLOv8+DDSC +CAM	9.57	12.45	85.21	15.01%	32.85%	5.73%
YOLOv8+DDSC +MLF YOLOv8+DDSC	9.35	12.01	86.14	16.96%	35.22%	6.89%
+CAM+MLF (DC-YOLO)	9.82	15.15	93.42	12.79%	18.28%	15.92%

To validate our method’s performance against other approaches, we selected Faster-RCNN (Wan and Goudos, 2020), YOLO-MECD (Liao et al., 2025), HPL-YOLOv4 (Xu et al., 2023), Yolo-FD (Lu et al., 2024), MFAF-YOLO (Zhang et al., 2024), ORD-YOLO (Huang et al., 2025b) as a comparison algorithm and conduct experiments using the same experimental environment and datasets. **Table 5** compares the detection accuracy, model scale and inference speed of DC-YOLO with mainstream two-stage and onestage detection algorithms. The two-stage Faster-RCNN exhibits the lowest precision and slowest frame rate with heavy parameters and computation. Among existing YOLO variants, ORD-YOLO achieves the lightest model size and highest FPS, while HPL-YOLOv4 obtains competitive mAP50 and mAP50:95 scores. Our DC-YOLO outperforms all competitors in Precision (0.980) and Recall (0.965), and achieves a mAP50:95 of 0.769, only slightly inferior to HPL-YOLOv4. In terms of efficiency, DC-YOLO has moderate parameters and its FPS of 93.42 is second only to ORD-YOLO. Overall, DC-YOLO strikes an excellent trade-off between detection accuracy, lightweight volume and real-time performance for citrus maturity identification in complex orchard scenes.

**Table 5:** Comparison of performance across different method.

Methods	Precision	Recall	mAP50	mAP50:95	#Params(M)↓	GFLOPs↓	FPS
Faster-RCNN	0.788	0.862	0.826	0.620	43.43	27.22	14.53
YOLO-MECD	0.854	0.889	0.872	0.680	8.42	20.65	21.72
HPL-YOLOv4	0.934	0.943	0.982	0.780	11.48	18.47	55.37
Yolo-FD	0.882	0.898	0.987	0.670	6.32	19.52	51.42
MFAF-YOLO	0.881	0.877	0.902	0.692	10.67	16.76	86.28
ORD-YOLO	0.938	0.916	0.969	0.650	6.29	14.16	126.13
DC-YOLO (Ours)	0.980	0.965	0.975	0.769	9.82	15.15	93.42

Red denotes the optimal value, and blue denotes the suboptimal value.

**Figure 7:**
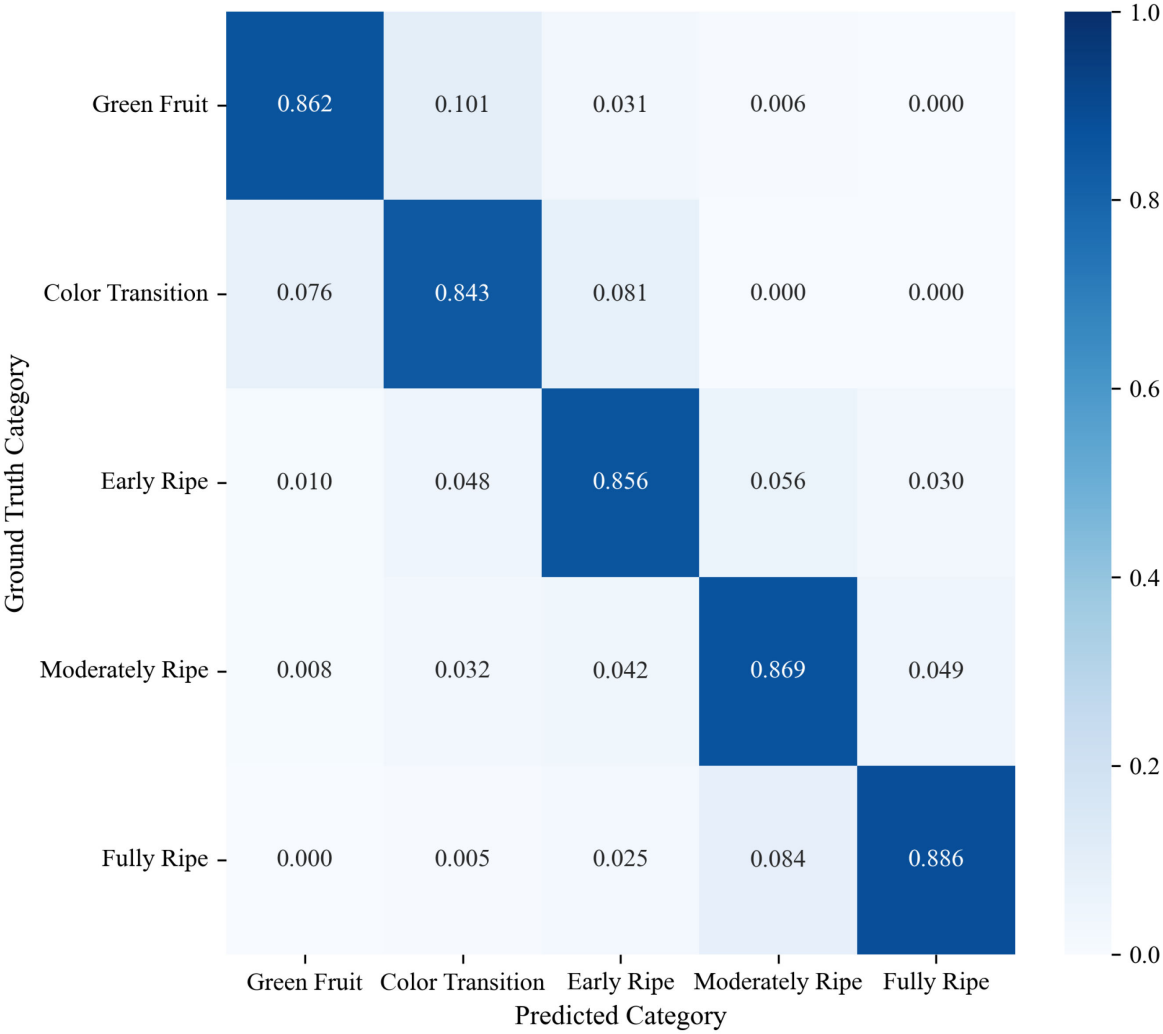
Ground truth normalized confusion matrix of citrus ripeness classification.

To further verify the accuracy of external citrus fruits at different levels of ripeness, a ground truth normalized confusion matrix was used to quantitatively assess the fine-grained classification performance of DC-YOLO across five maturity levels, as shown in **Figure 7**. Most misclassifications occurred only between two adjacent and similar classes, and the inter-class error rate was relatively low. There were virtually no cross-grade misclassifications between unripe and fully ripe fruits. These results validate that the proposed multi-loss function, by combining discrete classification with continuous ripeness regression, effectively mitigates the ambiguity of transitional citrus samples and enhances the model’s ability to distinguish subtle differences in peel color.

### 3.6 Model performance validation

**Figure 8:**
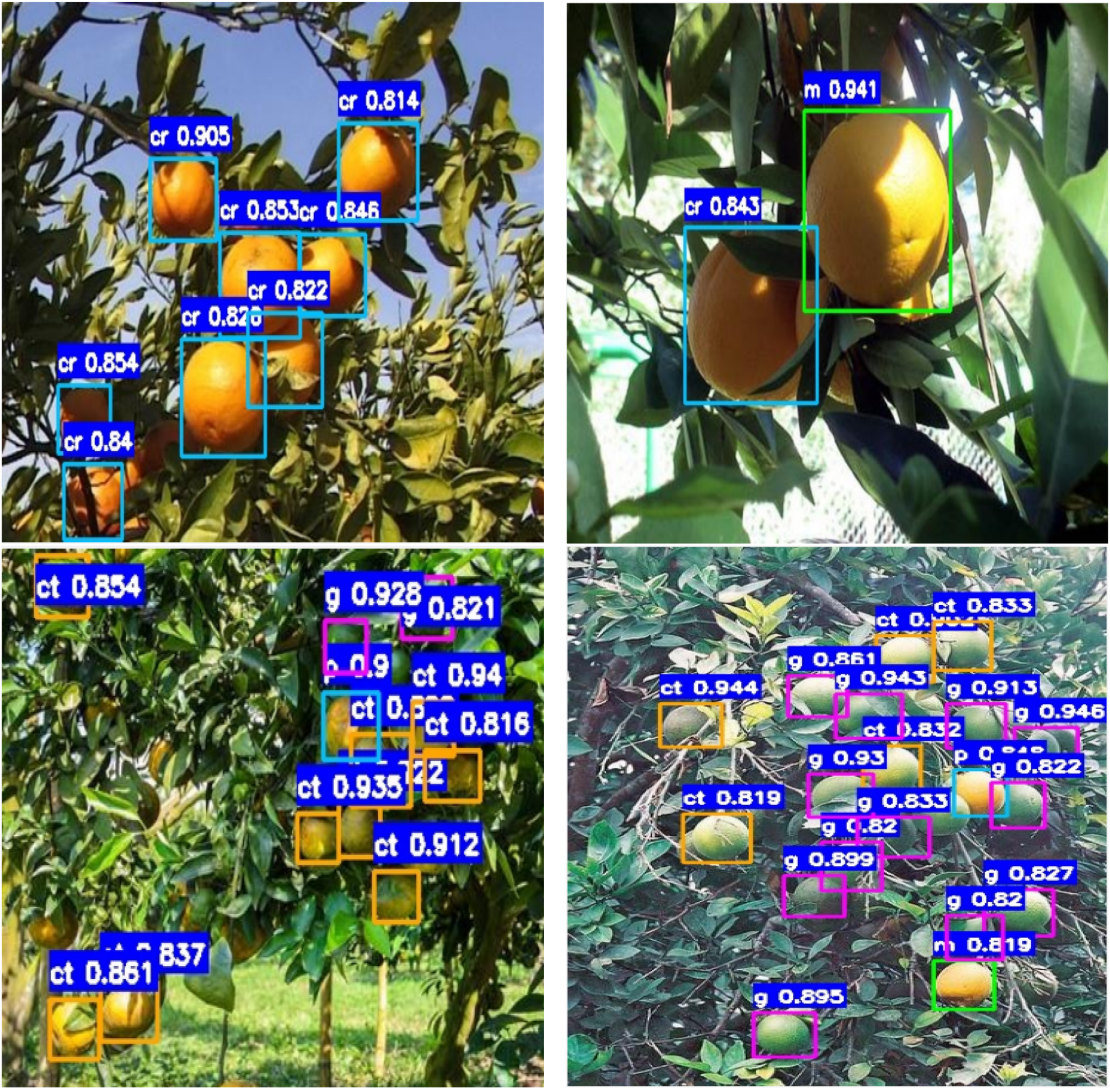
Results of field tests of this method in an orchard, where ‘g’ denotes the green fruit stage, ‘ct’ denotes citrus undergoing color transition, ‘p’ denotes primary ripe citrus, ‘m’ denotes moderately ripe citrus and ‘cr’ denotes complete ripe citrus, which corresponds to **Table 1**.

To qualitatively evaluate the model’s detection performance in complex orchard scenarios, we conduct a visual analysis of the inference results on the validation set, as shown in **Figure 8**, our model is capable of effectively handling complex scenarios such as fruit-leaf occlusion, overlapping fruits, and uneven lighting. For fruits of different sizes, the model achieved performance metrics of $AP_{small}$=0.876, $AP_{medium}$=0.927, and $AP_{large}$=0.962, validating the effectiveness of the introduced collaborative attention mechanism (CAM) and multi-loss function (MLF) in enhancing fine-grained discrimination capabilities and scene robustness.

**Figure 9:**
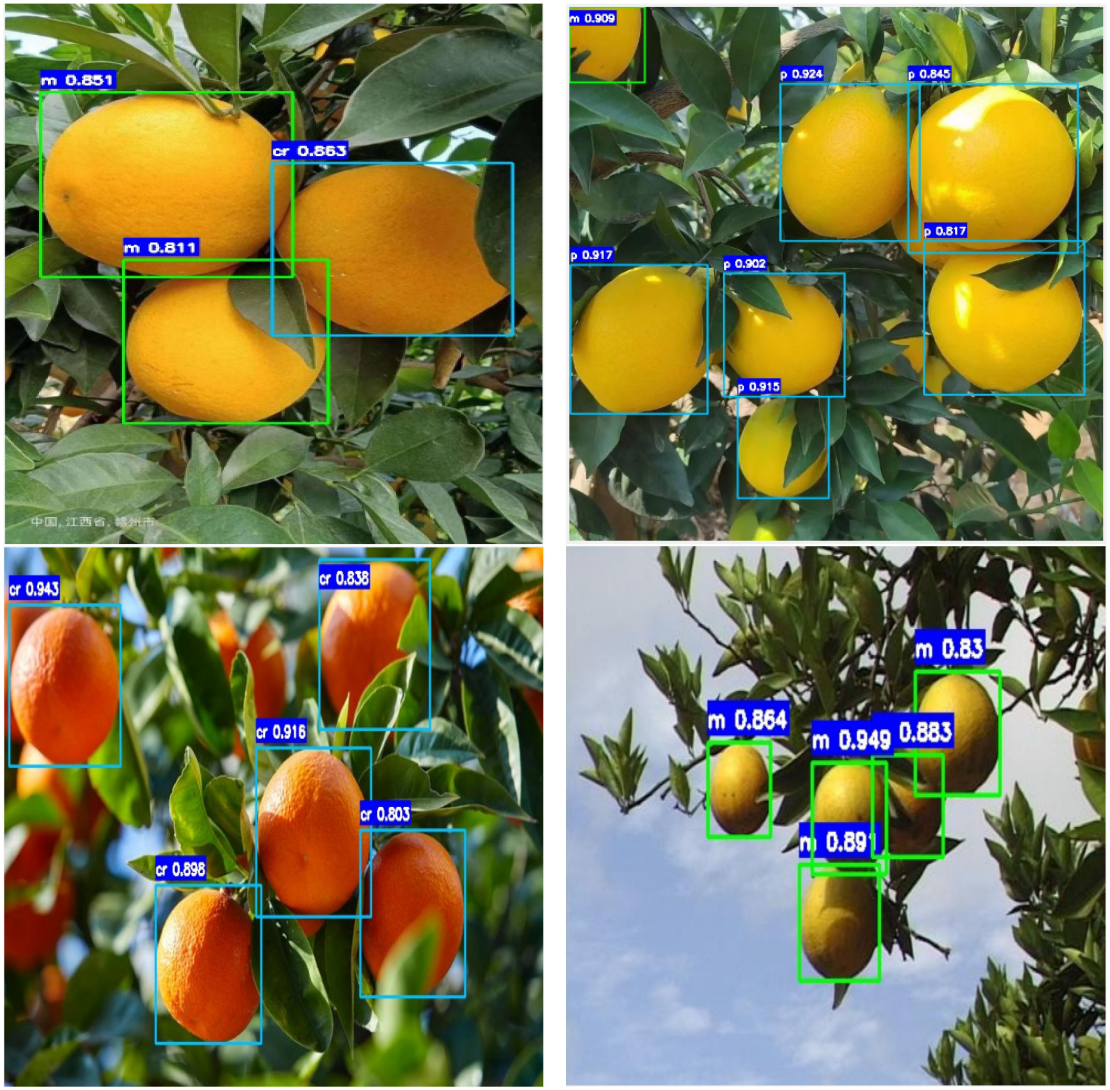
Actual results of citrus ripeness testing, where ‘g’ denotes the green fruit stage, ‘ct’ denotes citrus undergoing color transition, ‘p’ denotes primary ripe citrus, ‘m’ denotes moderately ripe citrus and ‘cr’ denotes complete ripe citrus.

This study uses images of citrus fruits collected from real orchards as its subject matter. By detecting the ripeness of citrus fruits in real-world orchard environment, the experimental results are shown in **Figure 9** under conditions involving varying lighting, occlusion, and background scenarios. Our model achieves an average detection accuracy of over 85% for citrus fruits at different stages of ripeness, thereby ensures the model’s generalization ability.

## 4 Discussion

This study aims to achieve accurate and efficient detection of citrus fruit ripeness in complex orchard environments. Based on the YOLOv8 framework, we developed an effective detection model suitable for citrus ripeness by integrating dynamic depth-separable convolutions, a collaborative attention mechanism, and multiple loss functions. Additionally, we constructed a citrus ripeness dataset covering different varieties at various stages of ripeness and across diverse natural scenarios, and enhanced sample diversity through data augmentation to provide reliable support for model training. Experimental results show that, compared to the original YOLOv8, the improved model achieves significant enhancements in both detection accuracy and fine-grained discrimination capabilities, while substantially reducing the number of parameters and computational load. Its detection frame rate meets real-time inference requirements, and the model effectively handles complex scenarios such as fruit-leaf occlusion and uneven lighting. The model performs exceptionally well in identifying fruits at the core maturity stage, with overall performance surpassing some existing methods in this field, thereby meeting the practical needs for accurate identification and intelligent harvesting in complex orchards. Despite the model’s advantages have been verified by extension experiments, DC-YOLO still has multiple non-negligible limitations in actual large-scale orchard monitoring and intelligent picking. The constraints in practical applications are as follows: (1) Limitation of discrimination between adjacent ripeness stages. Even under the joint constraint of classification and regression loss, for external datasets, the inter-class misclassification rate between color transition and early ripe citrus still reaches about 15%. For fruits with extremely close peel pigment proportions, the model cannot capture ultra-fine texture differences, which restricts its application scenarios requiring high-precision graded picking. (2) The overall data volume is too small and datasets class imbalance limitation. The total number of images is only 430. The self-built multi-orchard datasets has inherent sample quantity differences among five ripeness grades: fully ripe raw samples only account for 10.7% of all original images. Although Focal loss alleviates the issue of class weight imbalance during training, it still results in a slight decrease in the AP score for small object detection. All sample expansion depends on synthetic data augmentation, lacking a large number of real natural rare fruit images collected in the field. (3) Edge-device deployment constraints. The inference speed of 93.42 FPS was tested on a high-performance desktop GPU. When deployed on low-power embedded devices such as the Raspberry Pi and low-cost industrial cameras without GPU acceleration, the multi-branch attention module consumes a significant amount of memory bandwidth, resulting in a noticeable drop in frame rate. (4) The generalization test set contains only 80 unseen orchard images from new planting areas; the limited number of cross-region and cross-variety samples cannot fully verify the model’s long-term stability under continuous, large-scale picking operations. The aforementioned limitations will also be the focus of our future research.

## Data Availability

The original contributions presented in the study are included in the article/**Supplementary Material**. Further inquiries can be directed to the corresponding author.
