# Supplementary material for: An effective citrus ripeness detection model for complex orchard scenarios
Source: Front Plant Sci. 2026 Jul 24;17:1911329. doi: 10.3389/fpls.2026.1911329 (PMC13447265; doi:10.3389/fpls.2026.1911329)
Supplement: Supplementary file 1 [file DataSheet1.pdf]

Supplementary Material

1 SUPPLEMENTARY DATA

2 SUPPLEMENTARY TABLES AND FIGURES

The weight coefficient λ_{vis} is not an empirical constant without verification. We determine its optimal value via grid sensitivity experiments over the range [0.0, 0.1, 0.3, 0.5, 0.7, 0.9], taking mAP50 and mAP50:95 on the test set as the evaluation metric. The experimental sensitivity results are shown in the table S1.

Table S1. Ablation results of different λ_{vis} weights

λ_{vis}	0.0	0.1	0.3	0.5	0.7	0.9
mAP50	0.877	0.906	0.938	**0.975**	0.913	0.885
mAP50:95	0.636	0.724	0.747	**0.769**	0.751	0.736

2.1 Figures

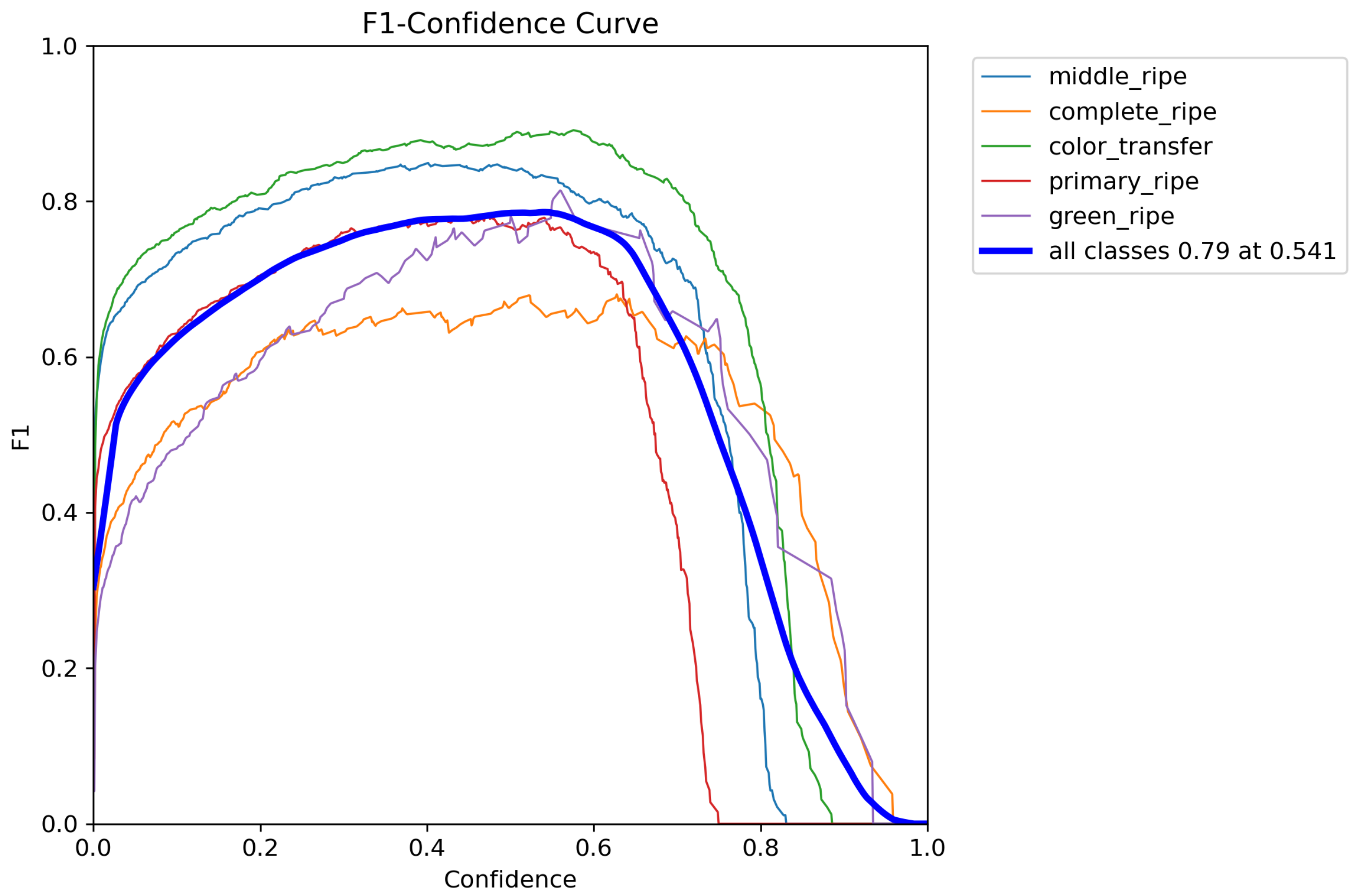

Figure S1. Confidence and F1 curve

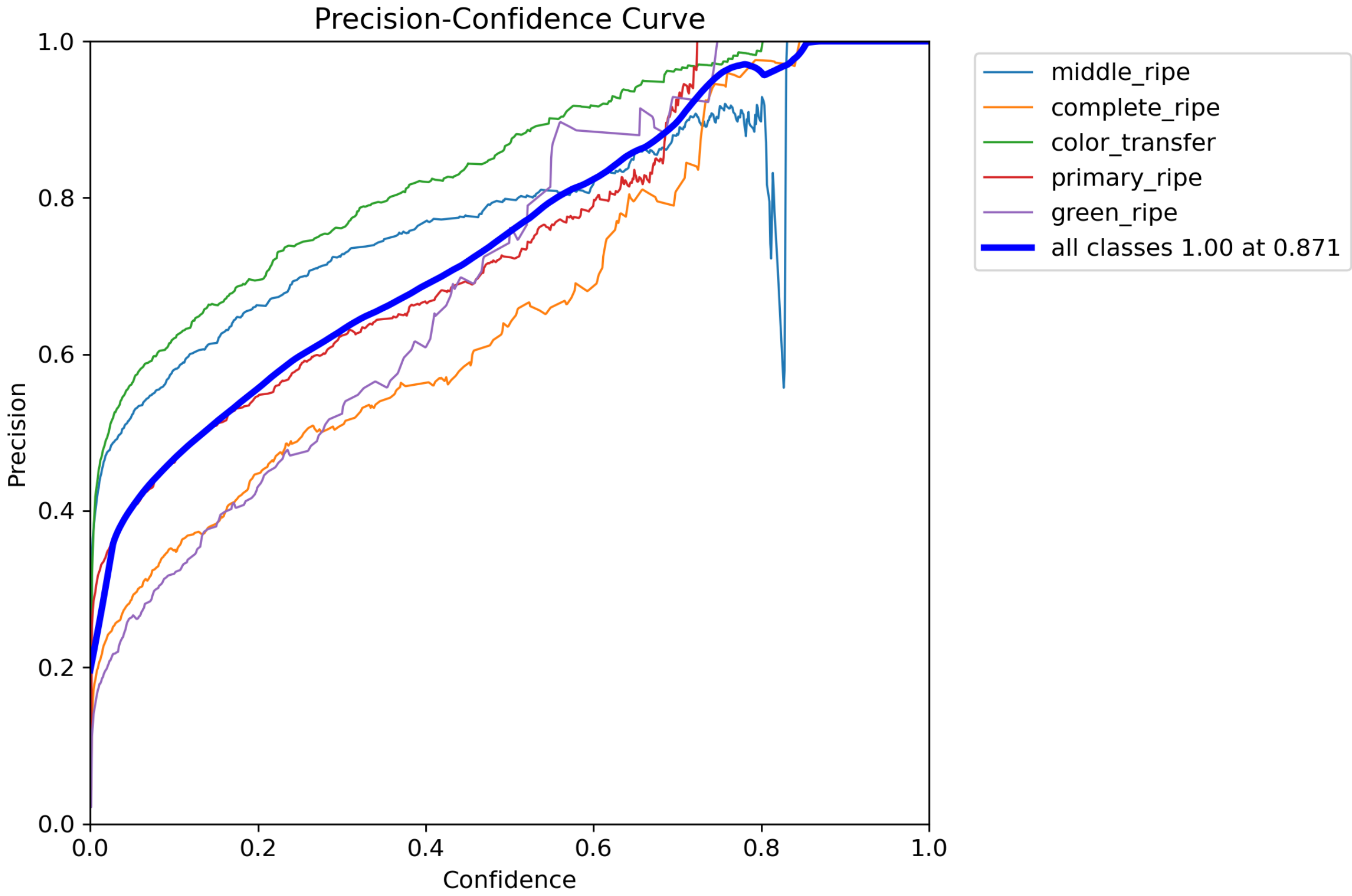

Figure S2. Confidence and Precision curve

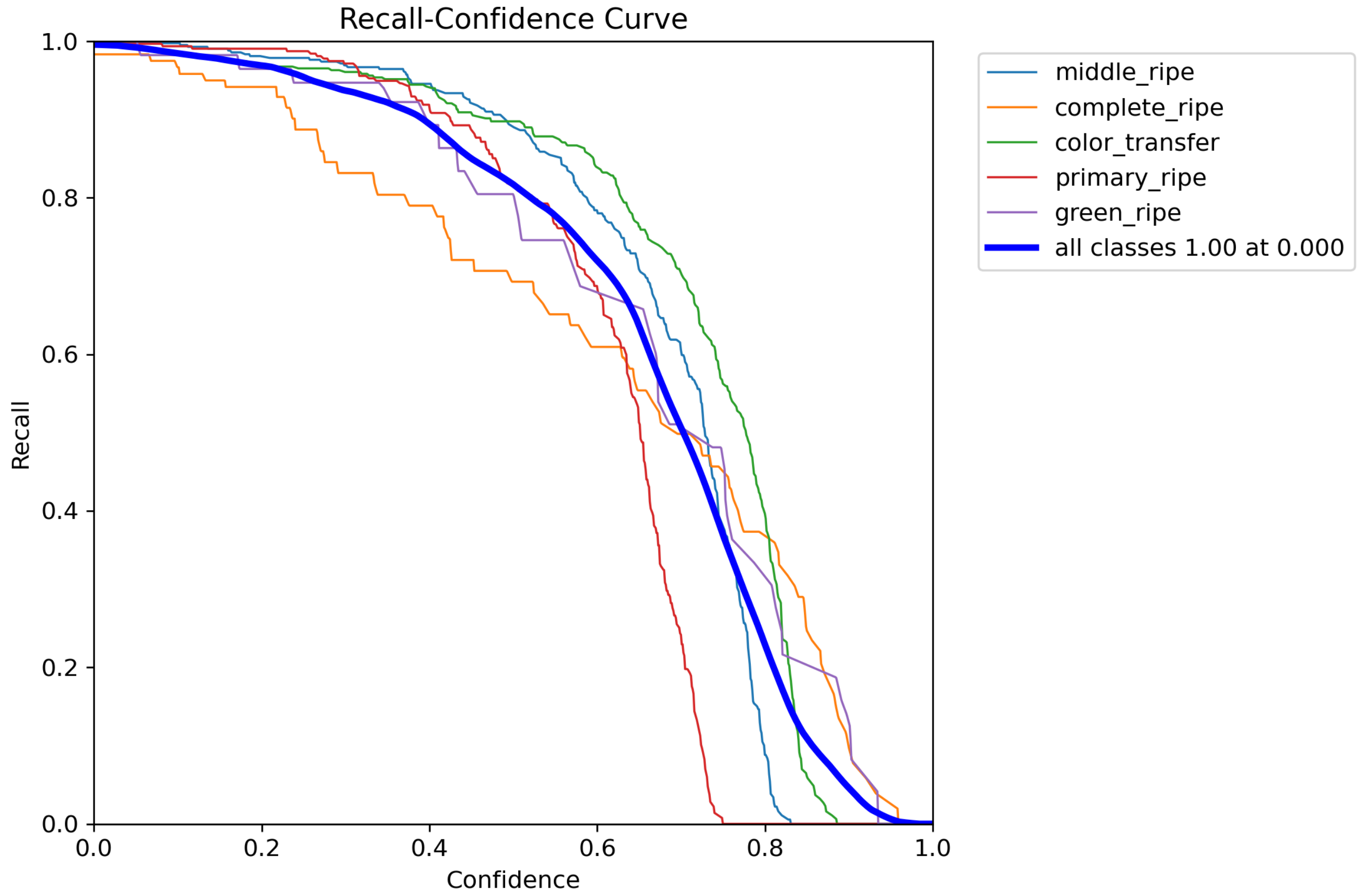

Figure S3. Confidence and Recall curve

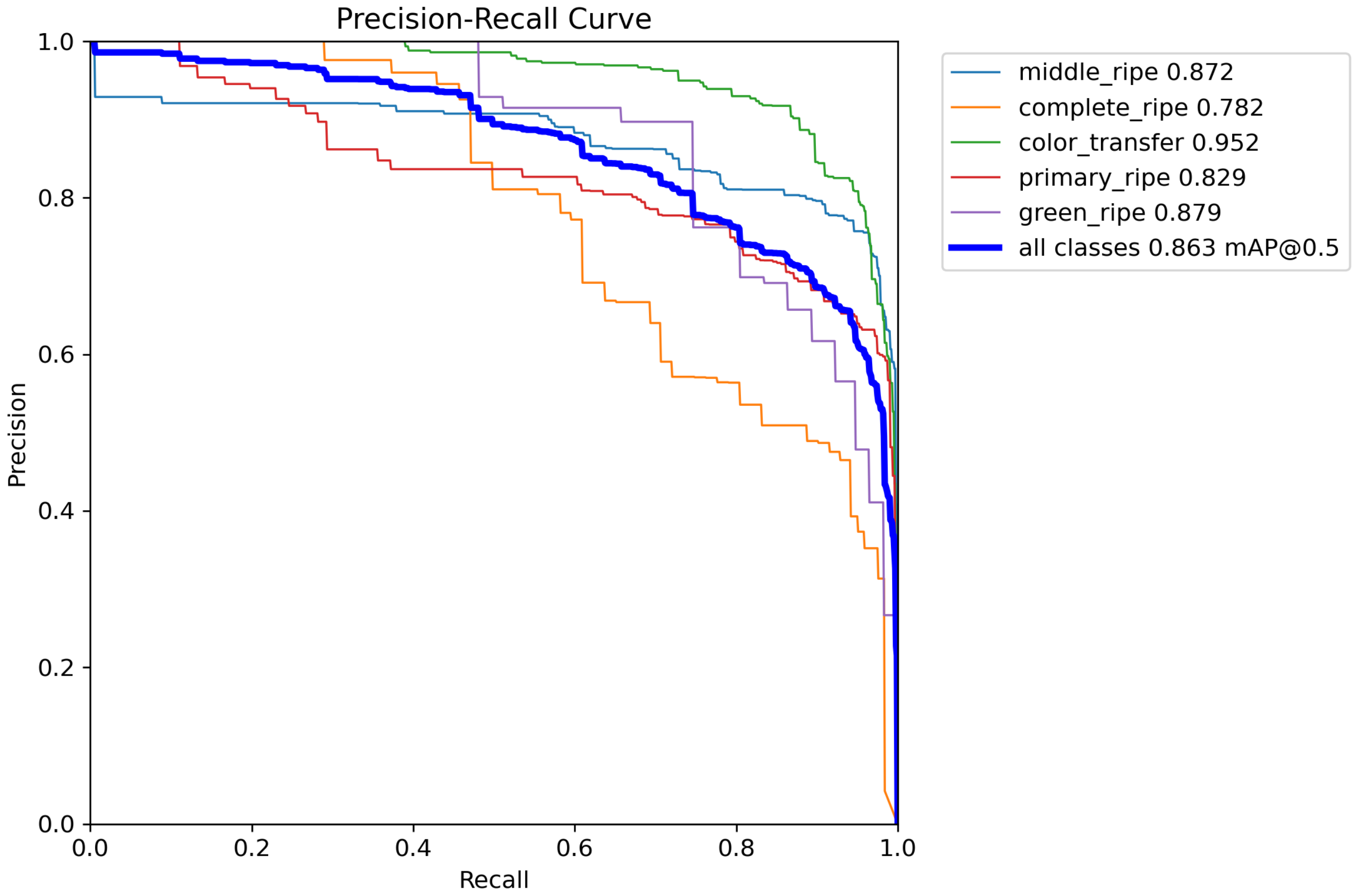

Figure S4. Recall and Precision curve
